# Steric ploy for alternating donor–acceptor co-assembly and cooperative supramolecular polymerization†
†Electronic supplementary information (ESI) available. See DOI: 10.1039/c6sc02640k



**DOI:** 10.1039/c6sc02640k

**Published:** 2016-09-19

**Authors:** Saptarshi Chakraborty, Haridas Kar, Amrita Sikder, Suhrit Ghosh

**Affiliations:** a Polymer Science Unit , Indian Association for the Cultivation of Science , Kolkata , India-700032 . Email: psusg2@iacs.res.in

## Abstract

The presence of a bulky peripheral wedge destabilizes the homo-assembly of an amide functionalized acceptor (A) monomer and thereby enables the formation of an alternating supramolecular copolymer with an amide appended donor (D) monomer *via* the synergistic effect of H-bonding and the charge-transfer (CT) interaction with a remarkably high *K*_a_ of 31 000 M^–1^. In sharp contrast, H-bonding driven homo-polymers of A and D are formed by just replacing the bulky chains of the A monomer with linear hydrocarbons. By taking advantage of the clear difference in the critical temperature for the onset of the AA or DD homo-assemblies and DA co-assembly (*T*_DA_ ≫ *T*_AA_ or *T*_DD_), the supramolecular polymerization pathway of the NDI-monomer could be fully diverted from isodesmic to cooperative in the presence of a small amount of DAN which helped the *in situ* production of nucleating sites involving the D–A CT-complex at a relatively higher temperature and the subsequent chain growth at *T*_AA_ following the nucleation-elongation model.

## Abstract 2

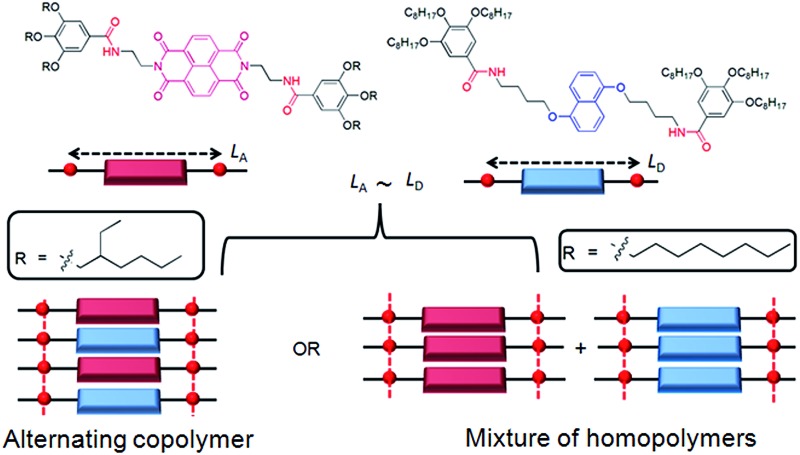
The presence of a bulky peripheral wedge destabilizes the homo-assembly of an amide functionalized acceptor monomer and thereby enables alternating supramolecular copolymerization with an amide appended donor monomer *via* the synergistic effect of H-bonding and the charge-transfer interaction.

## Introduction

**Scheme 1:**
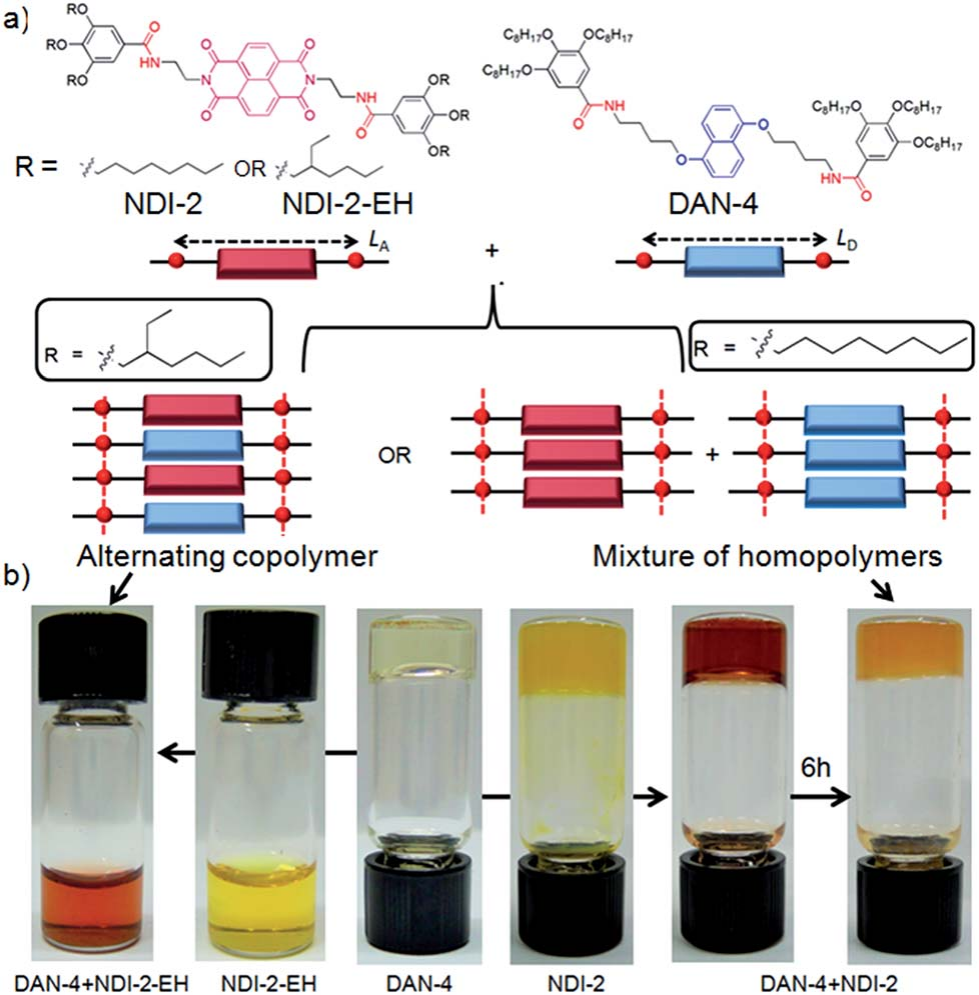
(a) Structure of DAN and NDI building blocks and peripheral alkyl chain dependent co-assembly. (b) Images of solution/gel in methylcyclohexane (*c* = 5.0 mM). The left and right arrows in the middle indicate transformation of DAN-4 gel to two different states when mixed with equal moles of NDI-2-EH and NDI-2, respectively. The right most arrow indicates the transformation of DAN-4 + NDI-2 (1 : 1) gel in methylcyclohexane from a mixed state to self-sorted state after 6 h upon standing at rt.

Organic donor (D)–acceptor (A) charge-transfer (CT)-complexes^1^ are of varied interest^2^ in the context of ambipolar charge transport,^3*a*^ ferroelectricity,^3b,c^ sensors,^3d,e^ ion channels,^3f–h^ photosystems,^3*i*^ thermochromic material,^3*j*^ foldamers,^3k,l,6a,b^ gels,^3m,n^ supra-amphiphiles,^3*o*^ rotaxane,^3*p*^ catenane^3*q*^ and other supramolecular systems.^2^ However, the weak association constants ranging between 1–20 M^–1^ for most D–A pairs^2^ is the major obstacle to achieving extended stacking as desirable in optoelectronic applications.^4^ Emerging reports though suggest hydrogen-bond^5*a*^ or halogen-bond^5*b*^ promoted co-assembly can construct mixed D–A co-crystals with long range order; the manifestation of such ordered arrays in solution remains an uphill task.^6^ Recently we have reported the H-bonding promoted co-assembly of bis-amide-functionalized naphthalene-diimide (NDI) acceptor or dialkoxy-naphthalene (DAN) donor units^7^ with matching spacer lengths (*L*_A_ and *L*_D_, respectively) between the two amide groups appended at either arm of the NDI and DAN chromophores. However even for the best match between *L*_A_ and *L*_D_ in NDI-2 + DAN-4 pair (Scheme 1a), the spontaneously formed CT-complex with intense red colour disappeared after ∼6 h (Scheme 1b) due to reorganization of the alternating D–A stack to individual homo-polymers of NDI and DAN.^8^ In a nutshell, while attempting to reinforce *via* H-bonding, we ended up fully sacrificing the inherent alternate stacking mode (though weak) of the DAN–NDI pair, which instead arrived at their self-sorted assembly.^3j,9,10,20^ This is because in the presence of the amide groups, the CT-interaction played a mere auxiliary role while the relatively stronger H-bonding preferred homo-assembly because in the alternating stack H-bonding had to be compromised due to the lack of a perfect match between *L*_A_ and *L*_D_. From the classical copolymer equation^11^ it is learnt that to achieve an alternating copolymer between two monomers (M_1_ and M_2_), their monomer reactivity ratios (*r*_1_ and *r*_2_, respectively) should be close to zero. By definition *r*_1_ = *k*_11_/*k*_12_, where *k*_11_ and *k*_12_ represent the rate constants of the reactions involving monomer M_1_ adding to M_1_ or M_2_, respectively. Likewise *r*_2_ = *k*_22_/*k*_21_ for M_2_. Therefore to meet the criteria of *r*_1_ ∼ 0 and *r*_2_ ∼ 0, *k*_11_ and *k*_22_ should be close to zero implying that the propensity of homo-polymerization should be negligible for both the monomers M_1_ and M_2_. Thinking along a similar line, we envisioned lowering the propensity of the homo-assembly of DAN and/or NDI might enable the formation of a stable alternating D–A copolymer. Notably, the value of *r*_DAN_ is a way lower as its homo-polymer is destabilized by electrostatic repulsion among the DAN chromophores.^8^ The challenge remains in lowering the value of *r*_NDI_ without jeopardizing the CT-complex formation. We envisaged the presence of branched peripheral alkyl chains selectively in the NDI building block (NDI-2-EH, Scheme 1) would destabilize its homo-assembly by steric repulsion. On the other hand in an alternating NDI–DAN stack, steric crowding will be less pronounced due to the buffering by the less space consuming linear peripheral alkyl chains of the DAN units.

## Results and discussion

**Fig. 1:**
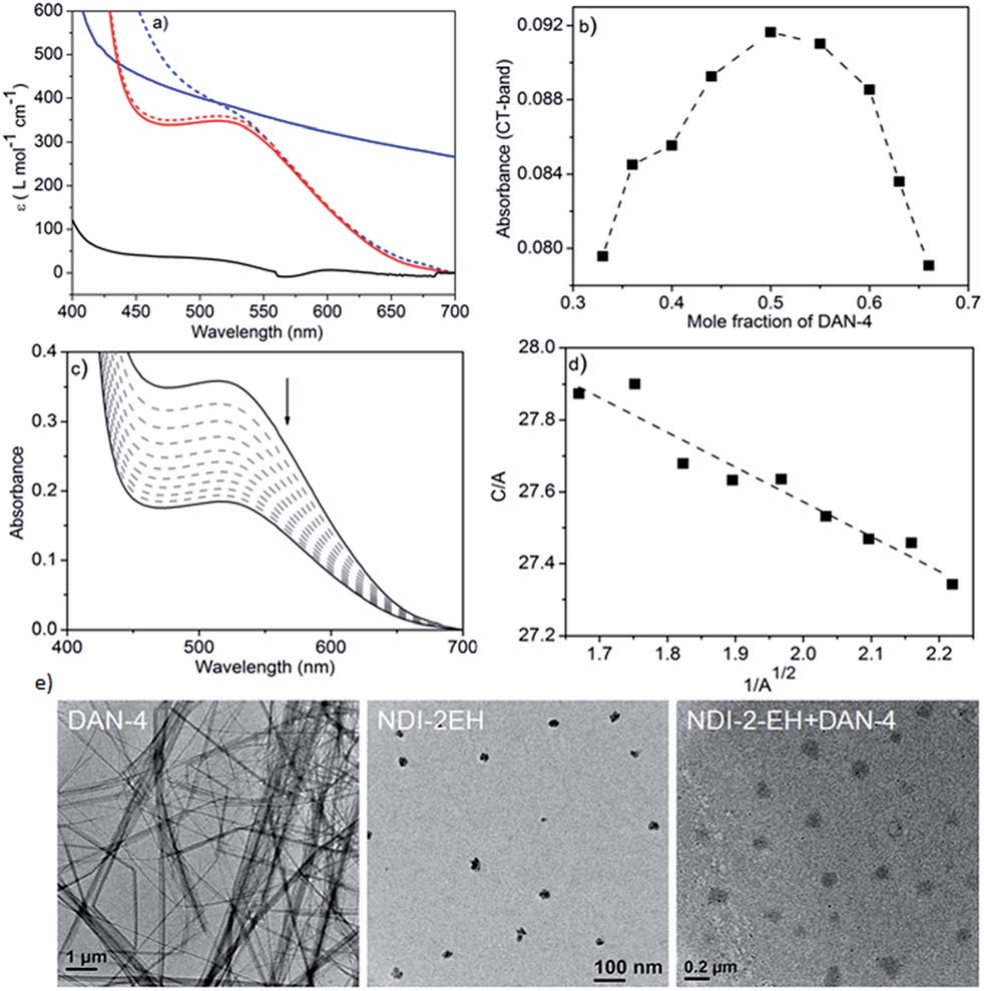
(a) UV/Vis spectra (selected section) of 1 : 1 DAN-4 + NDI-2-EH (red-dotted and solid lines show freshly prepared and aged samples after 30 days, respectively) and DAN-4 + NDI-2 (blue-dotted and solid lines show freshly prepared and aged samples after 6 h, respectively) in methylcyclohexane; black line shows DAN-4 + NDI-2-EH in CHCl_3_. In all experiments, the concentration of DAN = 10.0 mM and NDI = 10.0 mM. (b) Job's plot for DAN-4 + NDI-2-EH constructed from the CT-band intensity with different D/A ratios (total concentration fixed at 2.0 mM). (c) Concentration dependent UV/Vis spectra of DAN-4 + NDI-2-EH in methylcyclohexane (arrow indicates spectral change upon dilution) and (d) fitting of the data (eqn (1), ESI†) to estimate the association constant. *C* = 10 mM. (e) HRTEM images of different samples after drop-casting their methylcyclohexane solution on a carbon coated Cu grid.

To test this hypothesis we have studied the co-assembly of NDI-2-EH + DAN-4 (1 : 1) which produces an intense red solution in methylcyclohexane (Scheme 1) indicating an alternating D–A stack which is also evidenced by the appearance of a prominent CT-absorption band (Fig. 1a). Notably in chloroform, a good solvent, no red colour or any CT-band was visible (Fig. 1a) suggesting that co-assembly is prominent in less polarizable solvent.^4,7^ In sharp contrast, NDI-2 + DAN-4 (1 : 1) spontaneously forms a red gel but gradually the color disappears after 6 h (Scheme 1b) suggesting reorganization of the CT-state to a segregated state without disrupting the gel phase. It is interesting to note that the initial intensity of the CT-band for NDI-2 + DAN-4 gel is almost identical to that of NDI-2-EH + DAN-4 sol (Fig. 1a) indicating an equal population of the CT-complex at the beginning. However after 6 h, the CT-band fully disappears in the former case with a concomitant increase in the baseline intensity, possibly arising out of scattering due to the structural inhomogeneity of the self-sorted state.^12^ In contrast, for the NDI-2-EH + DAN-4 pair, no change in the CT-band intensity was noticed even after 30 days (Fig. 1a) ascertaining stable alternating co-assembly. Likewise the UV-range of the spectra (recorded at a lower concentration) also exhibits a significant change in its nature over time revealing reorganization of the chromophores from an initial assembled state to a different state after 6 h in case of NDI-2 + DAN-4, while a lack of such spectral changes in the case of NDI-2-EH + DAN-4 (even after 7 days) confirms a stable mixed assembly (Fig. S1†). Job's plots (Fig. 1b and S2†) confirmed a 1 : 1 complex which implies an alternating stack of the NDI and DAN chromophores. From the concentration dependent variation of the CT-band (Fig. 1c and d), the *K*_a_ was estimated (see ESI† for detailed calculation) to be 31 000 M^–1^ which is by far the largest value reported for a NDI–DAN pair and one of the very few higher values reported for any intermolecular D–A complex.^2^ Transmission electron microscopy (TEM) (Fig. 1e) images reveal a fibrillar network for DAN-4 which is typical for a gelator while the non-gelator NDI-2-EH shows spherical particles of diameter ∼ 25 nm.^13^ Interestingly for their mixture, the self-identity of the individual components are fully lost as neither any fibrils nor any small particles are visible anymore, rather the mixed assembly produced particles with a ∼100 nm diameter confirming the absence of individual homo-aggregates in the mixture. In a curiosity driven experiment, we placed a solution of NDI-2-EH in methylcyclohexane on top of a preformed DAN-4 gel while we noticed that gradually the red color evolved with concomitant transformation of the gel phase to a homogeneous solution (Fig. S3†) indicating that even the pre-formed homo-aggregates when in contact can reorganize to an alternating D–A copolymer.

**Fig. 2:**
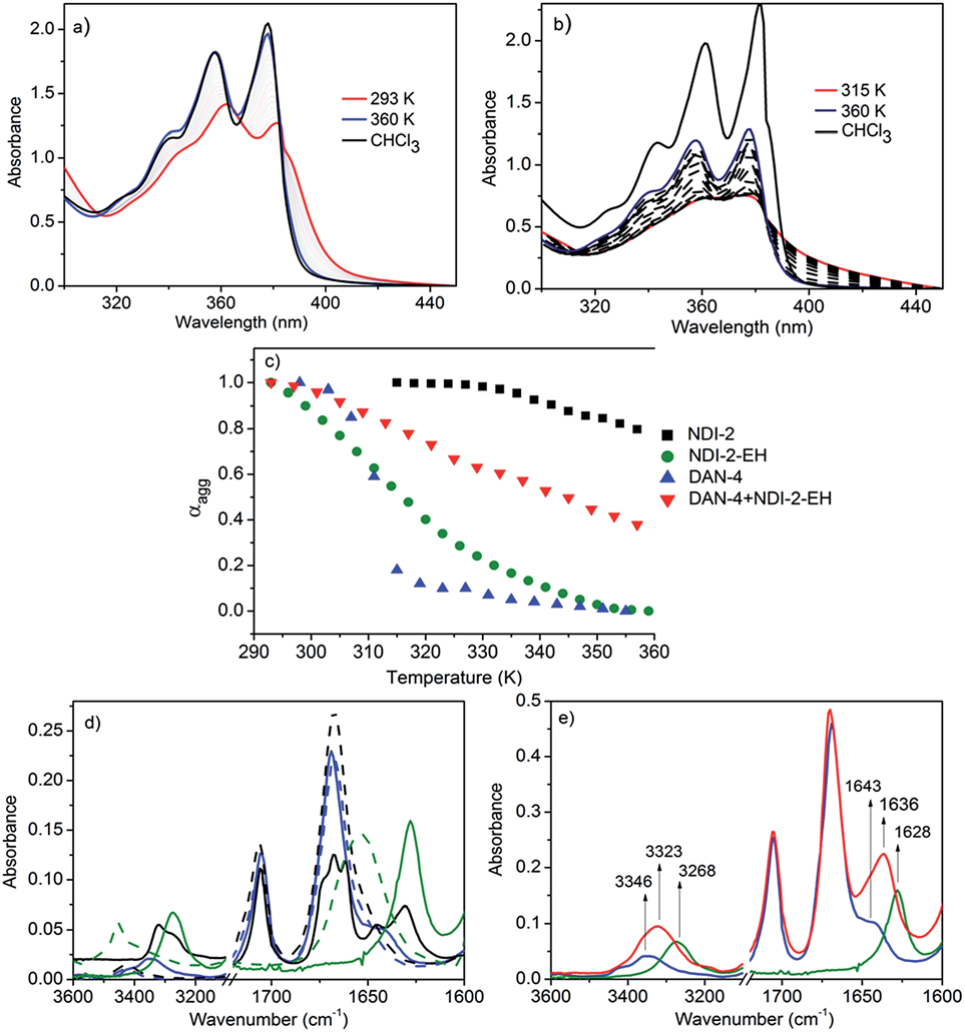
Variable temperature UV/Vis spectra of (a) NDI-2-EH and (b) NDI-2 in methylcyclohexane (*c* = 0.1 mM). (c) Melting curves for various self-assembling systems in methylcyclohexane. (d and e) Solvent dependent FT-IR spectra (dotted line-CHCl_3_, solid line-methylcyclohexane) of NDI-2-EH (blue), NDI-2 (black), DAN-4 (green) and NDI-2-EH + DAN-4 (1 : 1) (red).

UV/Vis spectra of NDI-2-EH and NDI-2 (Fig. 2) suggest monomeric species in CHCl_3_ and aromatic interaction in methylcyclohexane at rt. Temperature dependent studies (Fig. 2a and b) show a significant difference; for NDI-2 self-assembly is highly stable and even at the highest tested temperature the spectrum in methylcyclohexane does not resemble that of the monomer in CHCl_3_. On the other hand for NDI-2-EH at a higher temperature the spectrum matches that for CHCl_3_ suggesting complete disassembly. Melting curves (Fig. 2c) clearly indicate a much reduced stability of NDI-2-EH (compared to NDI-2) which is now comparable to even DAN-4 (Fig. 2c and S4†). Such a significant difference is attributed to destabilization of the NDI-2-EH homo-assembly due to steric repulsion among the peripheral chains as otherwise both chromophores are identical. But now the stability of the NDI-2-EH + DAN-4 (1 : 1) alternating stack is significantly enhanced compared to the homo-assembly of either component as both electrostatic and steric repulsion could be avoided in an alternating stack.

In the FT-IR spectra (Fig. 2d) both NDI-2 and NDI-2-EH show two distinct peaks at 1666 and 1706 cm^–1^ which are assigned^7^ to the symmetric and asymmetric stretching of the imide carbonyl, respectively. As no separate peak is noticed for the amide-1 band, it is assumed that in the non-H-bonded state (in CHCl_3_) it merges with the symmetric stretching peak of the imide carbonyl. In methylcyclohexane distinct new peaks emerge at 1630 and 1643 cm^–1^for NDI-2 and NDI-2-EH, respectively, due to the shift of the amide-1 band as a consequence of H-bonding. But the significant difference in the peak positions clearly indicates much weaker H-bonding for NDI-2-EH which corroborates with the observed shifts for the N–H stretching band (3418 to 3320 cm^–1^ and 3418 to 3346 cm^–1^ for NDI-2 and NDI-2-EH, respectively) and supports the melting curves (Fig. 2c). Weaker H-bonding is attributed to the steric crowding among the peripheral chains for NDI-2-EH. Likewise for DAN-4, the amide-1 band shows a significant shift from 1653 cm^–1^ to 1628 cm^–1^ and the N–H stretching band from 3452 cm^–1^ to 3268 cm^–1^ confirming strong H-bonding. Now for the mixture of NDI-2-EH + DAN-4 (Fig. 2e), distinct peaks appear at 1636 cm^–1^ and 3323 cm^–1^ for the amide-1 and N–H stretching bands, respectively, which indicate relatively stronger H-bonding compared to the NDI-2-EH homo-assembly owing to the release of the steric strain in an alternating stack. By comparing the FT-IR and UV/Vis data, it appears albeit forming strong H-bonds, the self-assembly of DAN-4 is least stable. It indicates in addition to H-bonding, aromatic interaction also contributes to the overall stability which is least beneficial for the homo-assembly of DAN but most beneficial for alternating NDI–DAN stacking. Overall the spontaneously formed CT-state shifts to thermodynamic products (H-bonding driven homo-aggregates) for NDI-2 + DAN-4 but remains stable for the NDI-2-EH + DAN-4 pair as in this case both the homopolymers are destabilized by steric and electrostatic repulsion, respectively.

**Fig. 3:**
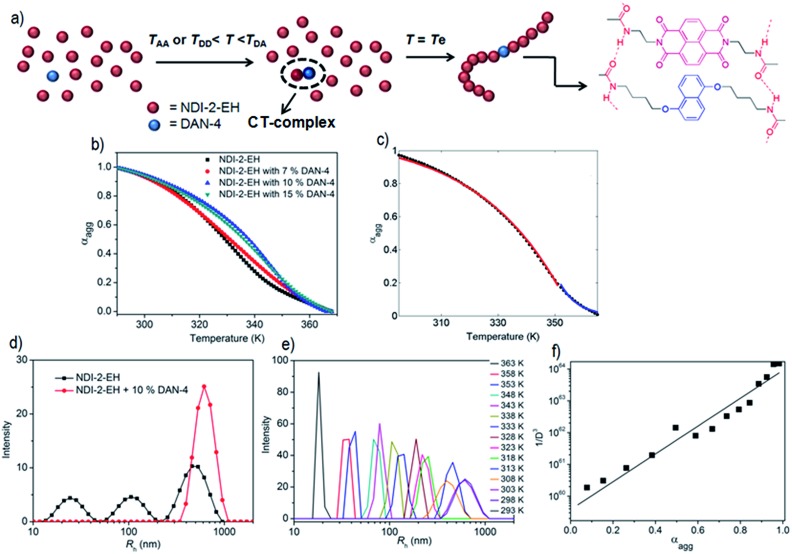
(a) Proposed model showing cooperative supramolecular polymerization of NDI-2-EH in the presence of 10% DAN-4. (b) Melting curve (derived from absorption at 395 nm) for NDI-2-EH in the absence and presence of DAN-4 in decane (*C* = 0.1 mM). (c) Fitting of elongation (red line) and nucleation (blue line) regime of the data for 10% DAN-4 in NDI-2-EH with eqn (1) and (2), respectively. (d) DLS data of supramolecular polymer formed by NDI-2-EH in the absence and presence of DAN-4. (e) Temperature-dependent DLS data for NDI-2-EH + 10% DAN-4 in decane and (f) the relationship of 1/*D* ^3^ (calculated from *R*_h_) with the mole fraction of the aggregate.

As an offshoot of the primary objective, we examined if such a kinetically trapped CT-complex could initiate cooperative supramolecular polymerization, which has captured immense attention in the recent past.^14^ We have already shown (Fig. 2c) that the temperature for the onset of homo-polymerization of NDI-2-EH (*T*_A_ ∼ 343 K) or DAN-4 (*T*_D_ ∼ 333 K) is significantly less compared to that for an alternating D–A stack (*T*_DA_ > 353 K). Therefore we envisaged that during cooling of a hot solution NDI-2-EH + DAN-4, when temperature would reach *T*_DA_, only DA or ADA type species would be generated by H-bonding assisted CT-interaction if *C*_A_ ≫ *C*_D_. Upon arrival at *T*_A_, the already formed CT-complex may act as the initiating site for the supramolecular polymerization^15^ of NDI-2-EH by a chain growth mechanism (Fig. 3a). To test this possibility we have probed the supramolecular polymerization of NDI-2-EH on its own and in the presence of 7–15% of DAN-4 by analyzing the nature of the respective cooling curves (Fig. 3b) generated from variable temperature UV/Vis studies. The cooling curve for NDI-2-EH fits well with the isodesmic model (Fig. S5†), similar to step-growth polymerization.^16^ But with 7% DAN-4 the curve starts deviating from a sigmoidal shape which becomes more prominent with 10% DAN-4 and does not change further with 15% DAN-4 (Fig. 3b). A much stiffer slope in the nucleation regime indicates cooperative self-assembly^17^ which was established by the satisfactory fit of the experimental data to the following eqn (1) and (2), representing the nucleation-elongation model.^18^
1
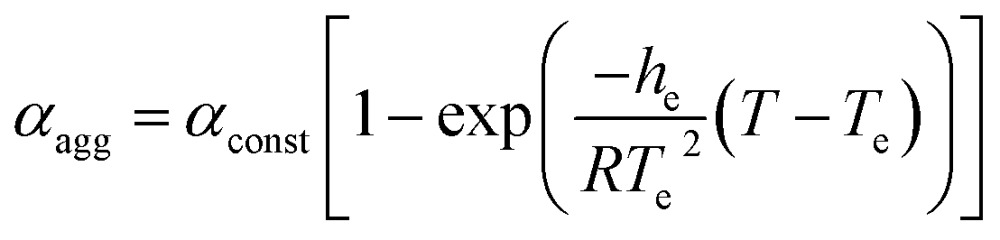

2
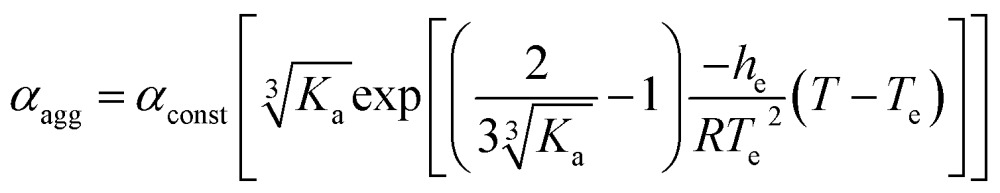

3
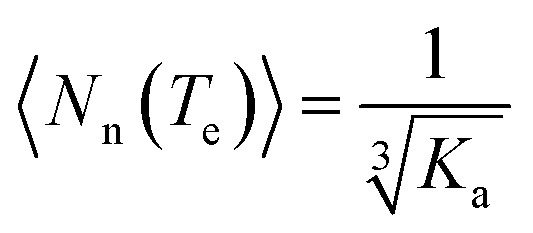
where *α*_const_ (used 1.100) is the parameter which assures *α*_agg_/*α*_const_ does not exceed unity, *h*_e_ is the molecular enthalpy released owing to non covalent interaction during elongation, *T* is absolute temperature, *T*_e_ is elongation temperature, *R* is ideal gas constant, *K*_a_ is the dimensionless equilibrium constant of the activation step at *T*_e_ and *N*_n_(*T*_e_) is the average length of the stack averaged over the nucleated species at *T*_e_. The nucleation and elongation regimes were treated independently with eqn (1) and (2), respectively, (Fig. 3c) revealing a very good fit. Dynamic light scattering (DLS) data of the supramolecular polymer of NDI-2-EH formed in the presence of 10% DAN-4 show a single peak (Fig. 3d) suggesting structural homogeneity while in the absence of any DAN-4, NDI-2-EH shows multiple peaks (Fig. 3d) with different particle size distributions reflecting uncontrolled polymerization. Further, temperature dependent DLS experiments show (Fig. 3e) the particle size gradually increases upon cooling a hot solution of NDI-2-EH in the presence of 10% DAN-4. The diffusion coefficients (*D*) were calculated at each temperature using the Stokes–Einstein formula (see ESI† for details) and as *D* varies inversely to the cube root of the molecular weight,^19^ we examined the relationship between the reciprocal of the cube of the diffusion coefficient with the *α*_agg_ at the corresponding temperature (which is similar to monomer conversion in conventional chain polymerization) which shows a linear relationship (Fig. 3f) as is typically observed for controlled chain polymerization. In contrast for NDI-2-EH alone, the temperature-dependent DLS data show the irregular variation and presence of multiple peaks (Fig. S6†) clearly suggesting distinct pathways for the supramolecular polymerization of NDI-2-EH in the presence and absence of DAN-4.

## Conclusion

This paper discloses two new findings: (i) a supramolecular strategy (based on steric factor) for stabilizing alternating donor (D)–acceptor (A) assembly and (ii) cooperative supramolecular polymerization of the A monomer only in the presence of a small amount of D, which helps in shaping the nucleus by an early formation of the CT-complex. While side chain (hydrocarbon, fluorocarbon or oligooxyethylene) immiscibility driven segregation^3j,20^ of π-systems or polymers is well known, it is less explored that two different hydrocarbons induce miscibility in an alternating sequence as demonstrated in this paper. This has far reaching consequences for controlling the sequence of dissimilar building blocks in multi-component self-assembly. Furthermore by taking advantage of the significantly higher stability of the alternating stack compared to individual homopolymers, it was possible to generate the CT-complex *in situ* in a mixture of A + D (*C*_A_ ≫ *C*_D_) at a relatively higher temperature, which acted as the nucleus for subsequent homo-polymerization of A *via* a cooperative mechanism in contrast to its isodesmic self-assembly in the absence of D. Unlike the majority of examples of nucleation involving the monomer itself,^14,17,21,22^ initiation by a distinct molecular entity^23^ (DAN-4) opens up a world of opportunities for supramolecular block-copolymer^24^ stereo-selective supramolecular co-polymerization (by using D and A building blocks with enantiopure side chains instead of a racemic mixture as used in the present study)^25^ and sequencing in precision supramolecular copolymers similar to emerging reports on aperiodic covalent copolymers.^26^
