# Supplementary material for: Steric ploy for alternating donor–acceptor co-assembly and cooperative supramolecular polymerization
Source: Chem Sci. 2016 Sep 19;8(2):1040–5. doi: 10.1039/c6sc02640k (PMC5356505; doi:10.1039/c6sc02640k)
Supplement: Supplementary file 1 [file SC-008-C6SC02640K-s001.pdf]

Electronic Supplementary Material (ESI) for Chemical Science.
This journal is © The Royal Society of Chemistry 2016

Supporting Information for

Steric Ploy for Alternating Donor-Acceptor Co-assembly and Cooperative Supramolecular Polymerization

Saptarshi Chakraborty, Haridas Kar, Amrita Sikder and Suhrit Ghosh*

[a] *Indian Association for the Cultivation of Science, Polymer Science Unit, 2A & 2B Raja S. C. Mullick*

**Corresponding author's email: psusg2@iacs.res.in*

Materials and Methods: All the reagents were received from Sigma Aldrich Chemical Co. and used without further purification. Solvents were purchased from commercial sources and purified by reported protocol.[1] Spectroscopy grade solvents were used for physical studies. ^{1}H NMR and ^{13}C NMR experiments were done on a Bruker DPX-400 MHz and 500 MHz NMR machine and all the data were calibrated against TMS. UV/ Vis absorption experiments were performed in a Perkin-Elmer Lambda 25 spectrometer. Mass spectrometric data were acquired by an electron spray ionization (ESI) technique on a Q-tof-micro quadruple mass spectrometer (Micro mass). FT-IR spectra were recorded in a Perkin Elmer Spectrum 100FT-IR spectrometer. Transmission electron microscopy (TEM) images were captured in a JEOL-2010EX machine operating at an accelerating voltage of 200 kV.

Synthesis and characterization: Synthesis of NDI-2 and DAN-4 has been reported by us earlier.[2-3] NDI-2-EH was prepared according to the scheme shown below.

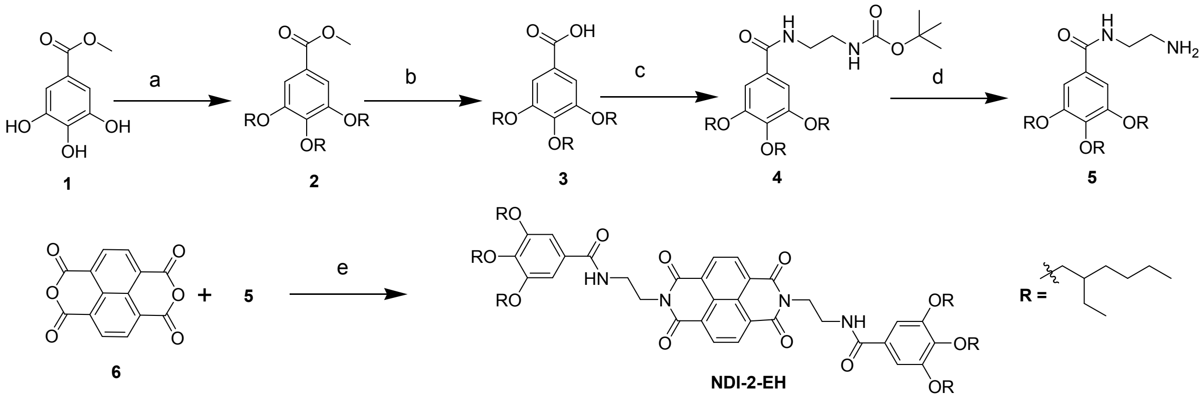

Reagents and conditions: a) 2-ethyl hexyl bromide, K_2CO_3, DMF, 100 ^{0}C, 48 h, 88%; b) KOH, EtOH/H_2O,5 h, 90 ^{0}C, 81% ; c) EDC, DMAP, CH_2Cl_2, rt, 12 h, 72% ; d) 4M HCl in Dioxane, rt, 4 h, 95% e) DMF, 140 ^{0}C, 24 h, 66%.

Scheme 1: Synthetic Scheme for NDI-2-EH.

Compound 2:[4] Compound **1** (1.0 gm, 5.43 mmol), 2-ethyl hexyl bromide (1.08 gm, 19 mmol), anhydrous K_2CO_3 (3.7 gm, 27.15 mmol) were taken together with 50 ml dry DMF and the reaction mixture was stirred at 100 °C for 48 h under N_2 atmosphere. The reaction was stopped, cooled to rt and poured in 100 mL water and the product was extracted with ethyl acetate (3 x 30 mL). The combined organic layer was washed with water (3 x 10 mL) followed by brine (1 x10 mL) and dried over anhydrous Na_2SO_4. Excess solvent was evaporated to get the crude product as brown oil which was further purified by column chromatography using basic alumina as a stationary phase and 2%

ethyl acetate-hexane as eluent to get the pure product as colorless oil. Yield: 1.9 gm (68%). ^{1}H NMR (400 MHz, $CDCl_3$, TMS): 7.27 (s, 2H), 3.94-3.86 (m, 9H), 1.79-1.33 (m, 27H), 0.82-0.78 (m, 18H).

Compound 3:[4] To a solution of compound **2** (1.0 gm, 1.92 mmol) in ethanol (10 mL), aqueous KOH solution (650 mg in 10 mL) was added and the reaction mixture was refluxed for 5 h at 90 ˚C. The reaction mixture was allowed to cool to rt and poured slowly into cold dil. HCl solution (50 mL). A white precipitate came out, which was filtered and washed with distilled water and dried under vacuum to get the crude product (**3**) as a colorless solid which was used for the next step without further purification. Yield: 800 mg (82%). ^{1}H NMR (400 MHz, $CDCl_3$, TMS): 7.32 (s, 2H), 3.94-3.89 (m, 6H), 1.78-1.26 (m, 27H), 0.93-0.89 (m, 18H).

Compound 4:[4] To a solution of compound **3** (500 mg, 0.98 mmol) in 10 ml dry CH_2Cl_2, 4-dimethylaminopyridine (23 mg, 0.19 mmol) and mono Boc-protected 1,2-diaminoethane (156 mg, 0.89 mmol) were added at 0 °C over 15 min. Subsequently, a solution of 1-Ethyl-3-(3-dimethylaminopropyl) carbodiimide (230 mg, 1.47 mmol) in CH_2Cl_2 (5 mL) was added drop wise to the reaction mixture. The mixture was stirred for 12 h at rt and then added with more CH_2Cl_2 (30 mL) and washed with HCl (1N) solution (3 x 10 mL) followed by brine (1 x 10 mL) and dried over anhydrous Na_2SO_4. Then excess solvent was evaporated to get the crude product. It was further purified by column chromatography using silica gel as the stationary phase and 30% ethyl acetate-hexane as the eluent to get the pure product (**4**) as a colorless solid. Yield: 450 mg (72%). ^{1}H NMR (400 MHz, $CDCl_3$, TMS): 7.41(s, 1H), 7.04 (s, 2H), 3.91-3.85 (m, 6H), 3.54 (t, J = 3.2 Hz, 2H), 3.38 (t, J = 3.4 Hz, 2H), 1.42 (s, 9H), 1.75-1.30 (m, 24H), 0.93-0.89 (m, 18H).

Compound 5:[4] Compound **4** (450 mg, 1.82 mmol) was dissolved in 10 ml 4.0 (M) HCl in dioxane and stirred at rt for 5 h. Then the solvent was evaporated under reduce pressure to get the white crude product. It was redissolved in CH_2Cl_2 (15 mL) and to this solution anhydrous K_2CO_3 (200 mg) was added and reaction mixture was stirred for 1 h, filtered and the volatiles were evaporated to get the product as colorless solid. It was taken to the next step without further purification. Yield: 360 mg (95%). ^{1}H NMR (400 MHz, $CDCl_3$, TMS): 7.46 (s, 1H), 7.04 (s, 2H), 3.86-3.81 (m, 6H), 3.62 (t, J = 3.3 Hz, 2H), 3.01 (t, J = 3.5 Hz, 2H), 1.29-1.26 (m, 24H), 0.93-0.89 (m, 18H).

NDI-2-EH: Compound **5** (350 mg, 0.63 mmol) and 1,4,5,8-napthalenetetracarboxylic-bisanhydride (86 mg, 0.31 mmol) were dissolved in dry DMF (10 mL) and refluxed at 140 °C for 24 h under N_2 atmosphere. After that the reaction mixture was allowed to cool to rt and placed in the refrigerator for 2 h while the crude product came out as an orange solid; which was filtered, and the obtained solid was washed with MeOH several times. The product was further purified by column chromatography by using silica gel as stationary phase and 1% MeOH in $CHCl_3$ as eluent to obtain the pure product as a light yellow solid. Yield: 280 mg (66%). ^{1}H NMR (500 MHz, $CDCl_3$, TMS): 8.72 (s, 4H), 6.90 (s, 4H), 6.75 (s, 2H), 4.53 (t, J = 5.9 Hz, 4H), 3.88-3.81 (m, 16H), 1.78-1.26 (m, 54H), 0.93-0.89 (m, 36H); ^{13}C NMR (100 MHz, $CDCl_3$): 167.8, 163.6, 153.3, 141.1, 131.2, 128.9, 127.0, 126.7, 105.0, 77.3, 76.1, 71.5, 40.7, 40.4, 39.9, 39.8, 30.7, 30.6, 29.8, 29.4, 29.2, 24.0, 23.8, 23.2, 14.2, 11.3, 11.2; LRMS (ESI): m/z calc for $C_{80}H_{120}N_4O_{12}$ $[M+Na]^+$: 1351.87; found: 1351.10; M.P: 139-142 ˚C.

Description on physical studies

Gelation Study: Stock solutions of all components were made in $CHCl_3$ at 10 mM concentration. Measured volume of aliquot was transferred to a screw capped vial and the solvent were evaporated by air blowing. Measured amount of methylcyclohexane was added to the vial to make the concentration of solute 10 mM and then the solutions were heated to make homogenous solution and allowed to rest at rt. Approximately after 5-10 min, NDI-2 and DAN-4 formed gel as tested by stable-to-inversion method whereas NDI-2-EH or DAN-4 + NDI-2-EH (1:1) remained as free flowing solution.

Alternating supramolecular copolymerization: Equal volume of an aliquot of DAN-4 and NDI-2-EH in $CHCl_3$ were mixed in a screw capped vial, solvent was evaporated and the red solid obtained was redissolved in measured volume of methylcyclohexane by heating which upon cooling to rt produced a red solution. The solution was allowed to equilibrate at rt for 2 h prior to carry out to any experiment. For estimation of association constant, the red solution was gradually diluted with a known volume of methylcyclohexane and CT-band (λ_{max} = 510 nm) was monitored as a function of concentration (10-5.5 mM). After addition of solvent, the solution was made homogenous and settled for 5 min before recording the absorption spectrum was recorded. K_a was estimated by fitting the experimental data to the following equation.[5]

$$\frac{C}{A} = \frac{1}{\sqrt{K\varepsilon l}} \times \frac{1}{\sqrt{A}} + \frac{1}{\varepsilon l}$$

Where c, A, l and Ɛ indicates concentration, absorbance, optical path length and extinction coefficient, respectively.

HRTEM experiments: Solution of DAN-4, NDI-2-EH and NDI-2-EH + DAN-4 (1:1) in methylcyclohexane (5.0 mM) were diluted to 0.1, 0.2 and 0.5 mM respectively and 10 µL of each solution was drop casted on a copper grid and dried at room temperature for 12 h before capturing images.

FT-IR Study: Stock solutions (5 mM) of NDI-2, NDI-2-EH, DAN-4 and NDI-2-EH + DAN-4 were prepared in both chloroform and methylcyclohexane solvents and spectra were recorded at room temperature.

Variable temperature UV-Vis studies: Solution of a given self-assembled chromophore(s) in methylcyclohexane (0.1 mM) was heated from 20 °C to 90 °C using a peltier attached to the UV/Vis machine and spectra were recorded at regular interval. From the temperature-dependent absorption spectra the mole fraction of aggregate at a given temperature T [$\alpha_{Agg}(T)$] was estimated using following equation.

$$\alpha_{Agg} = \frac{A(T) - A_{Mon}}{A_{Agg} - A_{Mon}}$$

Where A_{Mon}, A(T), and A_{Agg} are the absorbance at particular wavelength (326 nm for DAN-4, 377 nm for NDI-2 or NDI-2-EH and 505 nm for DAN-4 + NDI-2-EH) for the monomer (the value was taken from the absorption spectrum of the solution in $CHCl_3$), the solution in methylcyclohexane at temperature T and the fully aggregated stage (lowest temperature spectrum), respectively. $\alpha_{Agg}(T)$ was plotted as a function of T in each case to generate the melting curves shown in Figure 2c.

Supramolecular polymerization by nucleation-elongation pathway: Stock solution of NDI-2-EH and DAN-4 in $CHCl_3$ were mixed with appropriate ratio so that the NDI-2-EH concentration remained fixed while that for DAN-4 varied from 7-15% w. r. t. to the NDI-2-EH. Solvent was evaporated and to the solid measured amount of *n*-decane was added so that [NDI-2-EH] = 0.1 mM in each case. Absorption at a single wavelength (395 nm) was monitored as function of temperature (368 K to 290 K at 1 K/ min cooling rate) by Perkin-Templab software connected to the UV/Vis machine. The cooling curves were attempted to fit either the isodesmic or cooperative[5]. In isodesmic model, we used boltzman function, growth sigmodial in origin 8.0 software. After fitting the data of NDI-2-EH, we obtained correlation coefficient 0.9998. Whereas, the cooling curve of NDI-2-EH in presence of different percentage of DAN-4 failed to fit in isodesmic model. Thus we attempted to fit our data in cooperative model using gnu-software. First we separated elongation and nucleation regime and by using two known equations data was fitted as per reported literature.[6-7] Following a similar procedure, growth of supramolecular polymer was also monitored by temperature dependent (363-293 K, 5 K/ min) DLS studies which showed gradual increase in particle size. From these data diffusion coefficient (D) at each temperature was calculated using Stokes Einstein formula (shown below).

$$D = \frac{kT}{6\Pi \eta R}$$

Where *D, k*, *T*, п, ɳ, R stand for diffusion coefficient, Boltzmann constant, temperature, pi constant, specific viscosity of the solvent and hydrodynamic radius of the aggregates (measured by DLS). $1/D^3$ was plotted against α_{agg} at each temperature (estimated from cooling curve in UV/Vis experiment).

Additional Figures:

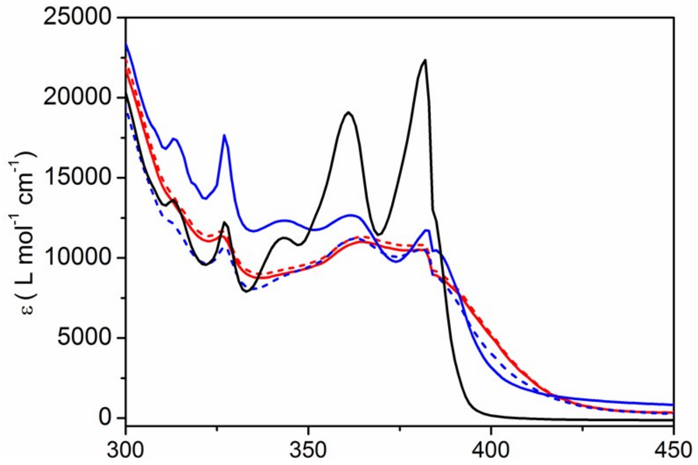

Figure S1: UV/Vis spectra of 1:1 DAN-4 + NDI-2-EH (red-dotted and solid line represent the spectra for the freshly prepared and aged sample after 7 days, respectively) and 1:1 DAN-4 + NDI-2 (blue-dotted and solid line show freshly prepared and aged sample after 6 h, respectively) in methylcyclohexane; Black line- 1:1 DAN-4 + NDI-2-EH in $CHCl_3$. In all experiments concentration of DAN =1.0 mM, NDI= 1.0 mM.

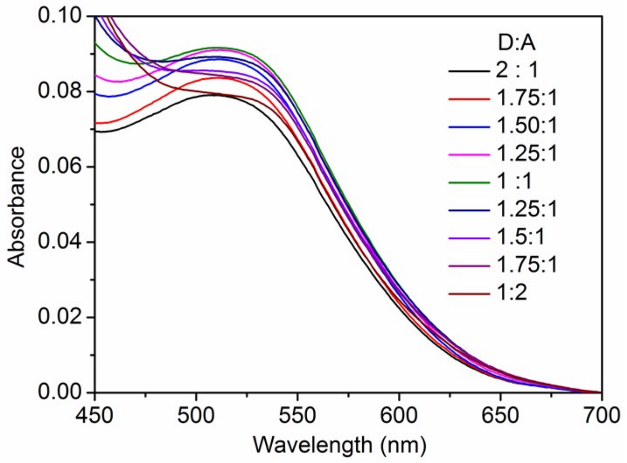

Figure S2: Stoichiometry dependent UV-Vis spectra of NDI-2-EH + DAN-4 in methylcyclohexane. c= 2 mM, l = 0.1 cm, T = 293 K.

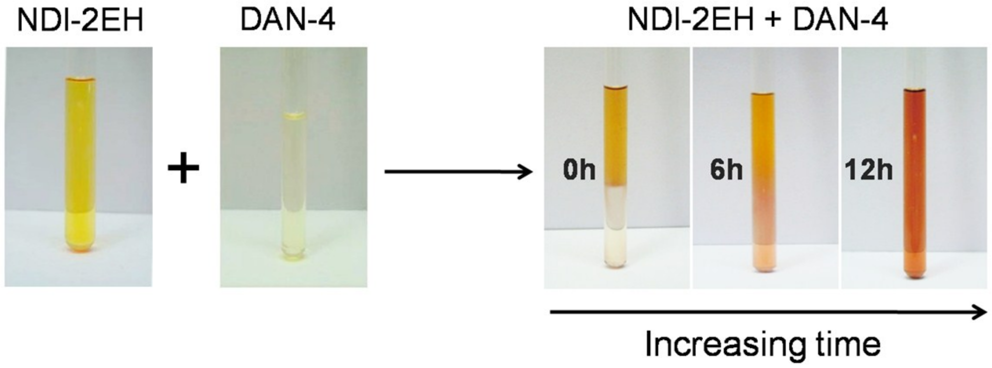

Figure S3: Pictures of (left) solution/ gel of NDI-2-EH and DAN-4 in methylcyclohexane (C = 10 mM) and (right) their fate after the solution of NDI-2-EH was added on top of the DAN-4 gel which eventually produced a red solution indicating alternating supramolecular polymerization.

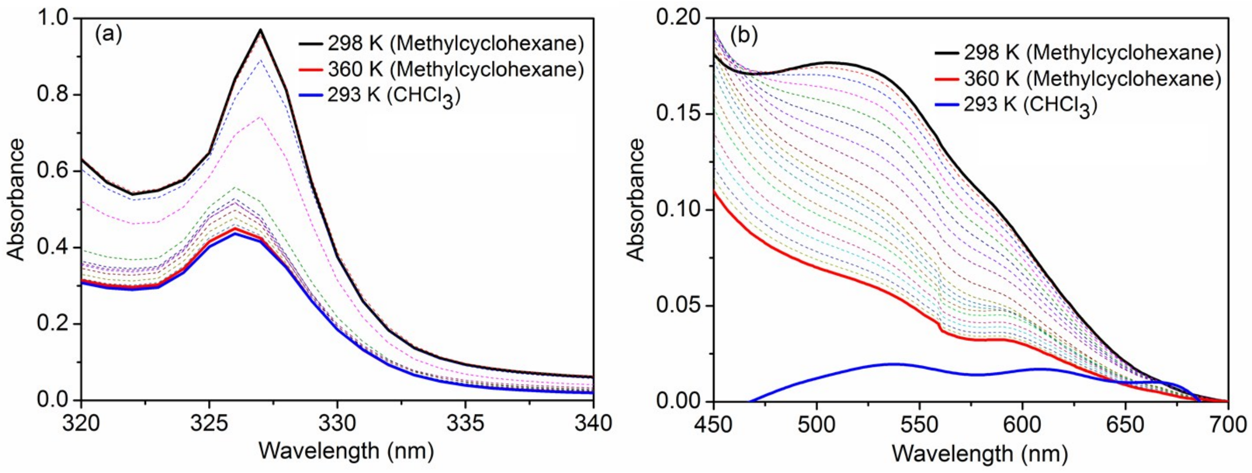

Figure S4: Variable temperature UV-Vis spectra of (a) DAN-4 and (b) NDI-2-EH + DAN-4 (1:1) in methylcyclohexane and the spectra in $CHCl_3$, c = 0.1 mM, l =1 cm.

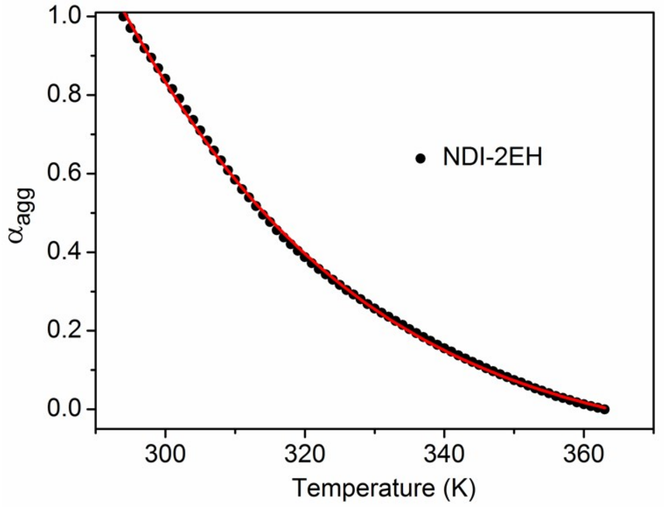

Figure S5: Fitting of the cooling curve of NDI-2-EH in decane by isodesmic model.

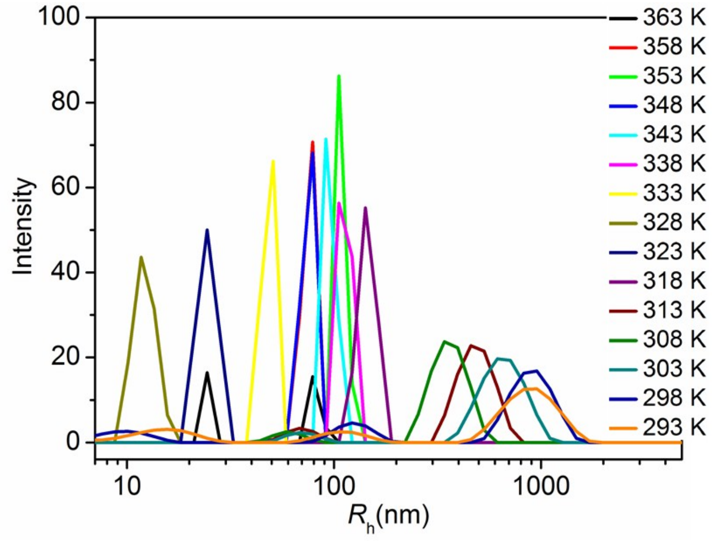

Figure S6: Variable temperature DLS of NDI-2-EH in decane (c = 0.1 mM) showing irregular growth and multiple peaks (particularly at lower temperature).

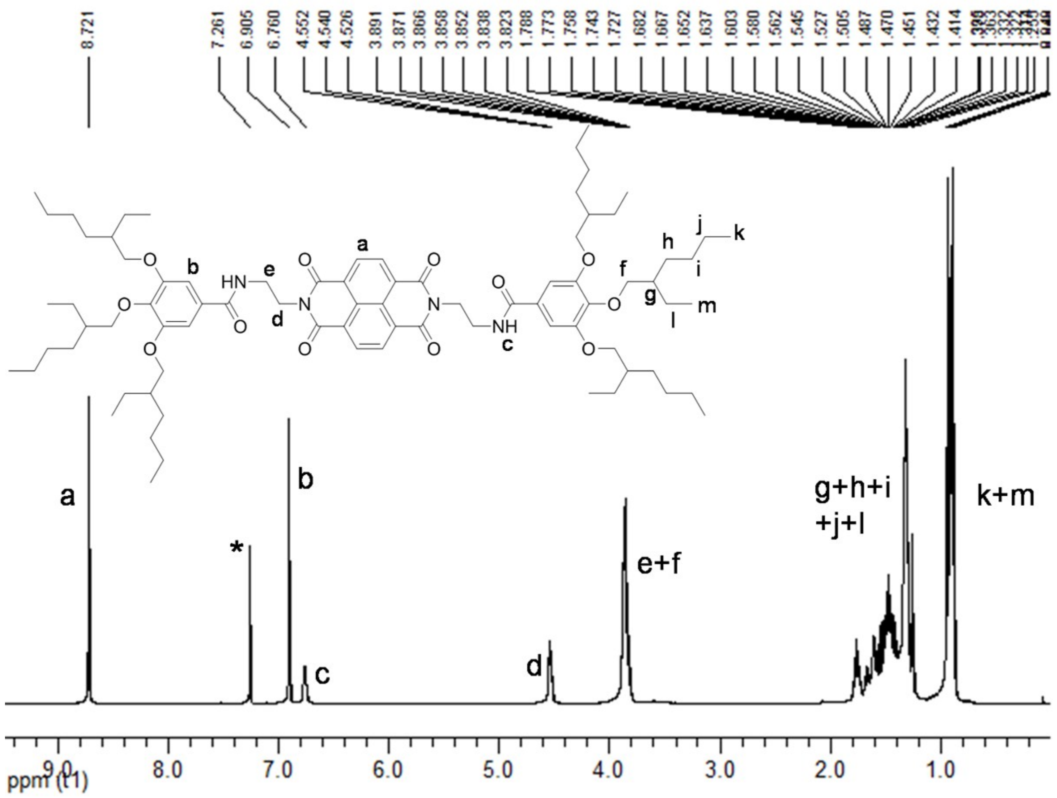

Figure S7: ^{1}H NMR spectra of NDI-2-EH in $CDCl_3$. * indicates residual solvent peak.

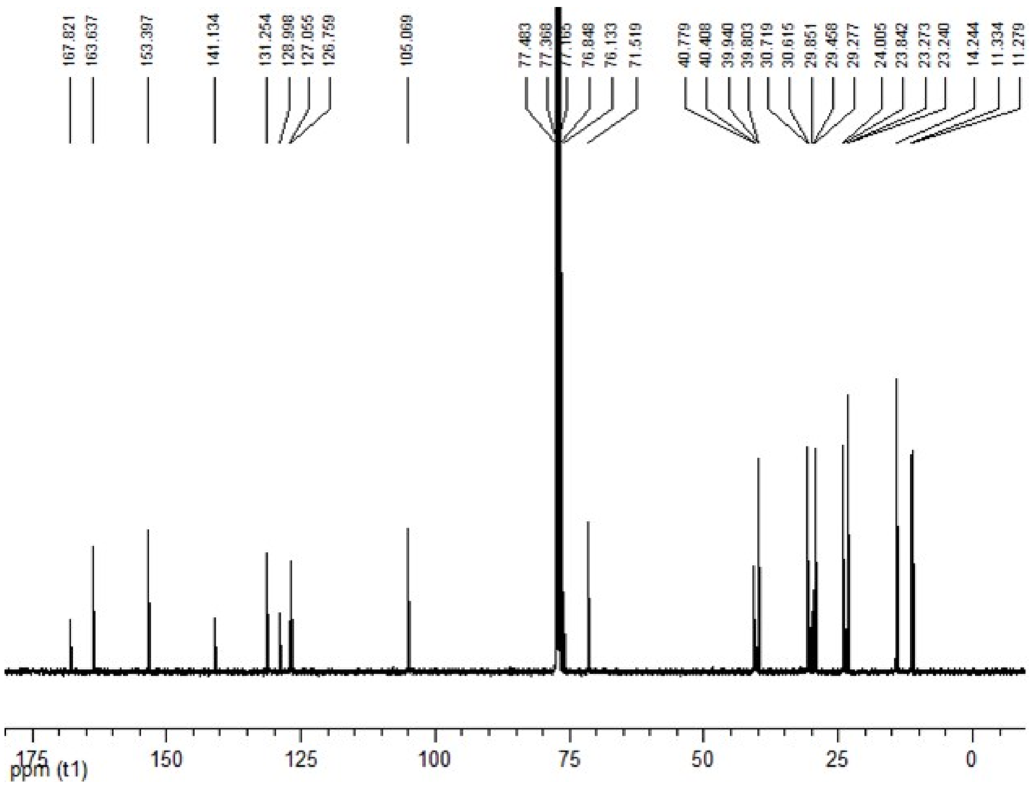

Figure S8: ^{13}C NMR spectra of NDI-2-EH in $CDCl_3$.

References:

1. D. D. Perrin, W. L. F. Armarego, D. R. Perrin, *Purification of Laboratory Chemicals*, 2nd ed.; Pergamon, Oxford, **1980**.
2. M. R. Molla and S. Ghosh, *Chem. Mater.*, 2011*,* **23***,* 95.
3. A. Das and S. Ghosh, *Chem. Eur. J.*, 2011*,* **17***,* 6061.
4. I. D. Tevis, L. C. Palmer, D. J. Herman, I. P. Murray, D. A. Stone and S. I. Stupp, *J. Am. Chem. Soc.*, 2011, **133**, 16486.
5. H. M. Colquhoun, E. P. Goodings, J. M. Maud, J. F. Stoddart, J. B. Wolstenholme and J. D. Williams*, J. Chem. Soc. Perkin Trans.*, 1985, **2**, 607.
6. M. M. J. Smulders, M. M. L. Nieuwenhuizen, T. F.A. de Greef, P. van der Schoot, A. P. H. J. Schenning and E. W. Meijer, *Chem. Eur. J*., 2010, **16**, 362.
7. P. Jonkheijm, P. van der Schoot, A. P. H. J. Schenning and E.W. Meijer, *Science*, 2006, **313,** 80.
